# LC–MS/MS Analysis Elucidates the Different Effects of Industrial and Culinary Processing on Total and Individual (Poly)phenolic Compounds of Piquillo Pepper (*Capsicum annuum* cv. Piquillo)

**DOI:** 10.1021/acs.jafc.2c07829

**Published:** 2023-04-04

**Authors:** Cristina Del Burgo-Gutiérrez, Concepción Cid, Iziar A. Ludwig, María-Paz De Peña

**Affiliations:** † Faculty of Pharmacy & Nutrition, Department of Nutrition, Food Science & Physiology, University of Navarra, 31008 Pamplona, Spain; ‡ Center for Nutrition Research, University of Navarra, c/Irunlarrea 1, 31008 Pamplona, Spain; § IdiSNA, Navarra Institute for Health Research, 31008 Pamplona, Spain

**Keywords:** (poly)phenols, bioactive compounds, antioxidants, Capsicum annuum, LC−MS/MS, industrial treatments, culinary treatments

## Abstract

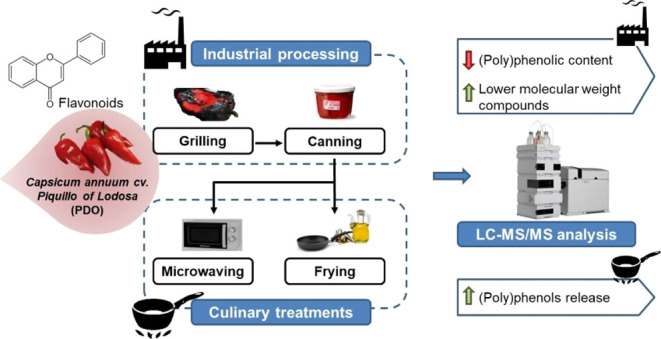

Pepper constitutes an important source of (poly)phenols, mainly flavonoids. Nevertheless, heat treatments applied prior to consumption may have an impact on these antioxidants, and thus may also affect their potential bioactivity. In this study, the effect of industrial and culinary treatments on the total and individual (poly)phenolic content of Piquillo pepper (*Capsicum annuum* cv. Piquillo) was thoroughly evaluated by high-performance liquid chromatography coupled to tandem mass spectrometry. A total of 40 (poly)phenols were identified and quantified in raw pepper. Flavonoids (10 flavonols, 15 flavones, and 2 flavanones) were the major compounds identified (62.6%). Among the 13 phenolic acids identified in raw samples, cinnamic acids were the most representative. High temperatures applied and subsequent peeling during industrial grilling drastically decreased the total (poly)phenolic content from 2736.34 to 1099.38 μg/g dm (59.8% reduction). In particular, flavonoids showed a higher reduction of 87.2% after grilling compared to nonflavonoids which only decreased by 14%. Moreover, 9 nonflavonoids were generated during grilling, modifying the (poly)phenolic profile. After culinary treatments, specifically frying, (poly)phenols appear to be better released from the food matrix, enhancing their extractability. Overall, industrial and culinary treatments differently affect both the total and individual (poly)phenolic compounds of pepper and, despite the reduction, they might also positively influence their bioaccessibility.

## 1 Introduction

Pepper (*Capsicum annuum*) is an herbaceous plant belonging to the genus *Capsicum* (*C.*) of the family *Solanaceae* and is cultivated worldwide in warm climate regions (northern and central America, southern and central Europe, Asia, and southern Africa).^1^*C. annuum* is the most produced and consumed of the five domesticated *Capsicum* species: *C. annuum*, *C. baccatum*, *C. chinense*, *C. frutescens*, and *C. pubescens*.^2−4^ In recent years, pepper has been suggested to play an important role in human health since it constitutes a rich source of bioactive compounds (carotenoids, vitamins, capsaicinoids, etc.), with increasing attention drawn to its (poly)phenolic content.^5−7^ Several epidemiological and clinical studies have evidenced a positive correlation between (poly)phenol consumption and a decreased risk of suffering from chronic diseases (cardiovascular diseases, cancer, diabetes, etc.).^8−12^ Besides its antioxidant activity, (poly)phenols’ anti-inflammatory properties have also been proposed as an underlying mechanism for improving the overall metabolic profile and the co-morbidities associated with chronic low-grade inflammation (glucose tolerance, hyperlipidemia, hypertension, etc.).^13,14^ Previous studies reported flavonoids as predominant compounds in different *C. annuum* varieties, mainly quercetin and luteolin derivatives, although cinnamic acids (caffeic, ferulic, *p*-coumaric, etc., and their derivatives) are also generally present.^7,15,16^

Piquillo pepper (*C. annuum* cv. Piquillo), accredited with European Protected Designation of Origin (PDO) recognition, is a distinctive variety of red pepper with unique organoleptic qualities that is grown in the south of Navarra (Spain) and considered one of the most representative products of their gastronomy. Piquillo pepper is generally commercialized in jars or cans, which involve two successive industrial heat treatments: a grilling technique at very high temperatures for a short period of time and subsequent peeling, followed by a canning process. Moreover, prior to consumption, canned pepper is typically submitted to different culinary processes. It is worth mentioning that heat treatments applied to vegetables in order to improve their edibility and palatability might either decrease their total (poly)phenolic content due to thermal degradation or increase it because of cell wall disruption, which leads to the release of bound (poly)phenolic compounds.^17−19^ Previous research on other vegetables (cactus cladodes, artichoke, and cardoon) reported that the cooking technique (time and temperature) along with the food matrix are the main factors affecting the total (poly)phenolic content and antioxidant capacity in vegetables.^5,18,20,21^ In this sense, the number of studies addressing the effect of cooking processes on pepper is limited, focusing mainly on drying processes and on Mexican chili pepper varieties.^7,22−27^ Moreover, only few studies have quantified (poly)phenolic content in pepper applying specific techniques such as liquid chromatography coupled to tandem mass spectrometry (LC–MS/MS), whereas the majority have performed Folin–Ciocalteu assays, known for overestimating the amount of (poly)phenolic content since other compounds such as vitamin C or protein carbonyls might also cause a reduction.^19^ To the best of our knowledge, only three studies have addressed the effect of culinary processes on *C. annuum* varieties by LC–MS/MS, specifically on red *cv. Aleppo and Capia*(5) and Italian green pepper.^28,29^ Nevertheless, Huarte et al.^29^ only evaluated this effect on digested pepper. Since the potential health benefits of plant foods depend not only on the total (poly)phenolic content but also on the individual compounds,^18,20^ the present study aimed to extensively evaluate the effect of industrial and culinary processes on the total and individual (poly)phenolic contents of Piquillo pepper (*C. annuum* cv. Piquillo) by HPLC–MS/MS.

## 2 Materials and Methods

### 2.1 Chemicals and Reagents

Methanol for LC–MS analysis was purchased from Panreac AppliChem (Darmstadt, Germany). Acetonitrile and 99% formic acid (both LC–MS grades) were obtained from Scharlau (Barcelona, Spain). The pure phenolic standards for LC–MS/MS were acquired from different manufacturers. In order to standardize, the recommended nomenclature of (poly)phenols proposed by Kay et al.^30^ is used in the present study (Table S1). Apigenin-7-*O*-glucoside, apigenin-8-*C*-glucoside, apigenin-6,8-*C*-diglucoside, luteolin, luteolin-7-*O*-glucoside, luteolin-8-*C*-glucoside, quercetin, quercetin-3-*O*-rutinoside, quercetin-3-*O*-glucoside, quercetin-3-*O*-rhamnoside, isorhamnetin, 4-caffeoylquinic acid, 5-caffeoylquinic acid, 4′-hydroxycinnamic acid, 3′,4′-dihydroxycinnamic acid, 4′-hydroxy-3′-methoxycinnamic acid, 3′-hydroxy-4′-methoxycinnamic acid, 4-hydroxy-3′,5′-dimethoxycinnamic, benzene-1,2-diol, 3-hydroxybenzoic acid, 4-hydroxybenzoic acid, 2,5-dihydroxybenzoic acid, 3,4-dihydroxybenzoic acid, and 4-hydroxy-3-methoxybenzoic acid were purchased from Sigma-Aldrich (Darmstadt, Germany). Standards of 3-(4′-hydroxy-3′-methoxyphenyl)propanoic acid and 3-(3′,4′-dihydroxyphenyl)propanoic acid were obtained from Alfa Aesar (Kandel, Germany). Naringenin-7-*O*-glucoside, kaempferol-7-*O*-glucoside, isorhamnetin-3-*O*-glucoside, and 2-(3′-hydroxyphenyl)ethanol, were acquired from Extrasynthese (Lyon, France).

### 2.2 Sample Preparation

Raw and thermally treated (grilled and canned) Piquillo peppers (*C. annuum* cv. Piquillo) were obtained from a local food industry in Lodosa, Spain. Raw Piquillo peppers were submitted to two successive industrial heat treatments, as stated in the PDO (grilling and canning).^31^ Additionally, canned peppers were submitted to two domestic culinary processes following the most common traditional culinary treatments in Navarra, Spain (microwaving and frying with olive oil).

Raw pepper samples were first washed to remove soil residue. Then, the core and the seeds were manually removed, and samples were chopped into small pieces prior to storage at −18 °C.

#### 2.2.1 Grilling

Whole raw peppers were industrially grilled at very high temperatures (ca. 700 °C) by direct flame for 15 s. Then, the core and peel of grilled samples were manually removed without water or any other solvents, as required under the PDO.^31^

#### 2.2.2 Canning

Grilled pepper samples were subsequently placed into jars with no brine addition and then submitted to commercial sterilization in canning retorts at 102 °C for 30 min.

#### 2.2.3 Microwaving

200 g of canned pepper were heated in a domestic microwave for 1 min at 750 W, covered with a microwave lid. This procedure was carried out twice, and both replications were mixed, obtaining a total of 400 g of microwaved Piquillo pepper.

#### 2.2.4 Frying

200 g of canned pepper were fried with 15 mL of olive oil in a nonstick frying pan for 6 min at approximately 90 °C. Then, samples were drained to remove the exceeding oil. This procedure was performed in duplicate, and both samples were mixed, obtaining a total 400 g of fried Piquillo pepper.

All samples were immediately cooled after cooking, and then raw and thermally treated samples were lyophilized in a freeze dryer, Cryodos-80 (Telstar, Terrasa, Spain). After lyophilization, each sample was ground into powder using a kitchen blender (La Moulinette 700 W, Moulinex, Alençon, France) and stored at −18 °C until further analysis.

### 2.3 (Poly)phenols’ Extraction

(Poly)phenolic compounds from raw and thermally treated pepper samples were extracted as described by Sánchez-Salcedo et al.^32^ with some modifications. Briefly, 25 mg of each ground sample were extracted with 0.5 mL methanol/acidified water (0.1% formic acid) (50:50, v/v) and then sonicated for 90 min in a sonic bath and centrifuged for 10 min at 14,000 rpm (Mikro 200. Hettich, Tuttlingen, Germany). The supernatant was collected, and the residue was re-extracted with 0.25 mL of methanol/acidified water (50:50, v/v); then, it was sonicated for 25 min and centrifuged a second time for 10 min at 14,000 rpm. The second supernatant was collected, and both supernatants were blended, filtered with a 0.22 μm PVDF syringe filter, and stored at −18 °C until LC–MS/MS analysis. The extraction procedure was performed in triplicate for each pepper sample.

### 2.4 Identification and Quantification of (Poly)phenolic Compounds

Qualitative and quantitative analysis of (poly)phenolic compounds in Piquillo pepper samples were carried out using a high-performance liquid chromatography (HPLC) unit model 1200 (Agilent Technologies. Palo 201 Alto, CA, USA) directly interfaced to a triple quadrupole linear ion trap mass spectrometer (3200 Q-TRAP LC–MS/MS) (AB SCIEX. Madrid, Spain), according to the method described by Domínguez-Fernández et al.^33^ with modifications.

For HPLC separation, mobile phase A was 0.1% (v/v) formic acid in water, and mobile phase B was acetonitrile. Chromatographic separations were achieved on a CORTECS C18 column (3 × 75 mm, 2.7 μm) from Waters (Barcelona, Spain), fitted in a column oven to operate at a controlled temperature (30 °C). The injection volume was 5 μL, and the elution flow rate was set at 0.6 mL/min. Gradient elution was as follows: 5% B (0–1 min), 5–10% B (1–5 min), 10–20% B (5–8 min), 20–14% B (8–8.5 min), 14–20% B (8.5–10.5 min), 20–30% B (10.5–16 min), 30–100% B (16–17.6 min), 100% B (17.6–25.6 min) 100–5% B (25.6–30.4 min) and maintained at 5% B until the end of the analysis (35 min). In order to determine (poly)phenolic compounds of Piquillo pepper, first a preliminary analysis was performed in a full scan operating mode, scanning *m*/*z* from 100 to 1000 followed up by a selective product ion mode (MS^2^) analysis. Finally, an ion multiple reaction monitoring analysis was carried out for the identification and quantification of (poly)phenolic compounds in Piquillo pepper samples.

Mass spectrometry analyses were run in the negative ionization mode, with the turbo heater operating at 600 °C and the ion spray voltage set at −3500 V. Nitrogen was used as nebulizing, turbo heater, and curtain gas and was set at −60, −65, and −35 psi, respectively. Declustering potential and entrance potential were set at −20 and −10 V, and collision energy (CE) for each compound was optimized using the same standards as for (poly)phenolic compound identification (Table S2).

Pure (poly)phenolic standards were used in order to identify (poly)phenolic compounds by comparing the molecular ion mass [M – H]^−^, retention time (*R*_t_), and MS/MS fragmentation. When no standards were available, (poly)phenolic compounds were tentatively identified by comparing MS/MS fragmentation with available literature and (poly)phenol databases (Human Metabolome Database, PubChem, and MassBank of North America). For quantification, individual calibration curves were built for each available pure standard. Tentatively identified compounds were quantified with calibration curves of the most structurally similar (poly)phenolic compounds. Luteolin-7-glucoside derivatives and luteolin-8-glucoside derivatives were quantified as luteolin-7-*O*-glucoside and luteolin-8-*C*-glucoside equivalents, respectively. Quercetin–glucoside, quercetin–rhamnoside, kaempferol–glucoside, and apigenin–glucoside derivatives were quantified as quercetin-3-*O*-glucoside, quercetin-3-*O*-rhamnoside, kaempferol-7-*O*-glucoside, and apigenin-7-*O*-glucoside equivalents, respectively. Glucosides of 3′,4′-dihydroxycinnamic acid, 4′-hydroxy-3′-methoxycinnamic acid, 4′-hydroxy-3′,5′-dimethoxycinnamic, and 4′-hydroxy-3′-methoxycinnamic acids were quantified as their respective aglycone equivalents. Finally, benzene-1,2,3-triol and 2′-hydroxy-4′-methoxyacetophenone were quantified as benzene-1,2-diol equivalents, 4′-hydroxy-3′-methoxyphenylacetic acid as 4′-hydroxy-3′-methoxybenzoic acid, 4-hydroxy-1,2-benzopyrone as 4′-hydroxycinnamic acid, and naringenin as naringenin 7-*O*-glucoside equivalents.

Chromatograms and spectral data were acquired using Analyst software 1.6.3 (AB SCIEX). Results were expressed in micrograms (μg) of (poly)phenol per gram (g) of pepper dry matter (dm). One way analysis of variance (ANOVA) was applied for each (poly)phenolic subgroup, and as a posteriori, the Tukey test was applied with a significance level of 95%. Both statistical analyses were carried out using the STATA v.12.0 software package. Principal component analysis (PCA) and heatmap analysis were performed using MetaboAnalyst 5.0. (https://www.metaboanalyst.ca/). For PCA, data were logarithmically transformed in order to deal with zeros and “pareto” scaled to reduce the relative importance of large values while staying closer to the original data. In the heatmap, compounds were hierarchically clustered according to the Ward clustering method and based on Euclidean distance.

## 3 Results and Discussion

### 3.1 Analysis of (Poly)phenolic Compounds of Raw Piquillo Pepper by HPLC-MS/MS

A total of 40 (poly)phenolic compounds were identified and quantified in raw Piquillo pepper (*C. annuum* cv. Piquillo) (Table 1). Flavonoids were the major compounds identified in raw pepper, within which 10 were flavonols (mainly quercetin derivatives), 15 flavones (predominantly luteolin derivatives), and 2 flavanones (naringenin derivatives). Thirteen phenolic acids were also identified in the raw samples, with cinnamic acids being the most representative (9 compounds). For the present research, the standardized nomenclature for (poly)phenols based on their molecular structure proposed by Kay et al.^30^ is used in order to ensure the correct interpretation of the results (Table S1). The mass spectrometric characteristics of (poly)phenolic compounds identified in Piquillo pepper are detailed in Supporting Information (Table S2).

**Table 1:** Concentration of the Main (Poly)phenolic Compounds in Raw and Thermally Treated Piquillo Pepper^a^

compound^c^		raw	grilled	canned	microwaved	fried
Nonflavonoids						
	benzenediols and triols					
**1**	benz-1,2-diol	nd	35.23 ± 1.24	28.50 ± 0.99	28.70 ± 0.25	25.15 ± 0.45
**2**	benz-1,2,3-triol^b^	nd	5.90 ± 0.02	3.93 ± 0.28	4.10 ± 0.08	4.74 ± 0.17
	total benzenediols and triols	nda	41.12 ± 1.22d	32.43 ± 1.27c	32.80 ± 0.29c	29.90 ± 0.53b
	benzoic acids					
**3**	3-OH-BA	nd	2.91 ± 0.03	0.65 ± 0.02	0.81 ± 0.04	0.71 ± 0.03
**4**	4-OH-BA	nd	0.88 ± 0.01	1.40 ± 0.03	1.27 ± 0.06	1.06 ± 0.09
**5**	2,5-diOH-BA	nd	0.49 ± 0.01	0.33 ± 0.03	0.36 ± 0.01	0.35 ± 0.03
**6**	3,4-diOH-BA	nd	1.61 ± 0.08	4.06 ± 0.12	3.93 ± 0.09	3.83 ± 0.02
**7**	3-MetOH-BA-4-*O*-GlucSD^b^	219.53 ± 5.61	200.31 ± 2.47	91.04 ± 5.11	98.78 ± 7.10	108.28 ± 4.63
	total benzoic acids	219.53 ± 5.61c	206.21 ± 2.55c	97.47 ± 5.15a	105.15 ± 7.11ab	114.23 ± 4.71b
	cinnamic acids					
**8**	4′-OH-CA	0.13 ± 0.00	0.61 ± 0.02	0.51 ± 0.01	0.61 ± 0.02	0.58 ± 0.05
**9**	CA-4′-*O*-GlucSD^b^	477.83 ± 17.94	221.61 ± 4.54	128.08 ± 0.72	136.27 ± 0.04	150.76 ± 9.06
**10**	3′,4′-diOH-CA	0.26 ± 0.02	0.15 ± 0.01	0.28 ± 0.00	0.27 ± 0.00	0.30 ± 0.00
**11**	4′-OH-CA-3′-*O*-GlucSD^b^	25.00 ± 2.35	14.06 ± 0.39	10.48 ± 0.81	11.06 ± 0.79	11.50 ± 0.60
**12**	4′-OH-3′-MetOH-CA	1.54 ± 0.09	1.33 ± 0.12	4.82 ± 0.07	4.27 ± 0.08	4.72 ± 0.08
**13**	3′-OH-4′-MetOH-CA	0.34 ± 0.01	0.33 ± 0.01	0.71 ± 0.02	0.72 ± 0.06	0.60 ± 0.04
**14**	3′-MetOH-CA-4′-*O*-GlucSD^b^	272.67 ± 20.72	189.21 ± 6.13	98.07 ± 4.77	96.23 ± 5.54	113.15 ± 6.19
**15**	4′-OH-3′,5′-diMetOH-CA	0.89 ± 0.08	0.95 ± 0.04	1.66 ± 0.01	1.60 ± 0.06	1.51 ± 0.05
**16**	3′,5′-diMetOH-CA-4′-*O*-GlucSD^b^	23.03 ± 0.37	11.95 ± 0.30	7.14 ± 0.68	7.40 ± 0.73	8.23 ± 0.07
	total cinnamic acids	801.70 ± 21.19d	440.19 ± 10.99c	251.76 ± 6.31a	258.43 ± 7.07ab	291.35 ± 15.42b
	phenylpropanoic acids					
**17**	3-(3′,4′-diOH-ph)PrA	nd	1.57 ± 0.01	1.27 ± 0.03	1.28 ± 0.04	1.14 ± 0.02
	total phenylpropanoic acids	nda	1.57 ± 0.01d	1.27 ± 0.03c	1.28 ± 0.04c	1.14 ± 0.02b
	phenylacetic acids					
**18**	4′-OH-3′-MetOH-phAc^b^	nd	187.83 ± 3.50	122.46 ± 5.55	117.33 ± 4.61	138.11 ± 6.77
	total phenylacetic acids	nda	187.83 ± 3.50c	122.46 ± 5.55b	117.33 ± 4.61b	138.11 ± 6.77bc
	other phenolic acids					
**19**	4-OH-1,2-BenzPyON^b^	nd	1.52 ± 0.04	1.07 ± 0.03	1.22 ± 0.09	1.19 ± 0.05
**20**	2′-OH-4′MetOH-Ac-phON^b^	1.41 ± 0.13	0.84 ± 0.01	0.37 ± 0.01	0.35 ± 0.03	0.45 ± 0.04
	total other phenolic acids	1.41 ± 0.13a	2.36 ± 0.03b	1.44 ± 0.05a	1.57 ± 0.11a	1.64 ± 0.09a
	acylquinic acids					
**21**	5-CQA	2.10 ± 0.04	1.24 ± 0.08	1.98 ± 0.09	1.96 ± 0.06	1.35 ± 0.02
**22**	4-CQA	tr	0.08 ± 0.01	0.20 ± 0.00	0.25 ± 0.01	0.17 ± 0.01
	total acylquinic acids	2.01 ± 0.03c	1.31 ± 0.08a	2.18 ± 0.09c	2.20 ± 0.07c	1.52 ± 0.02b
	total non-flavonoids	1.024.73 ± 26.86d	880.60 ± 14.65c	509.01 ± 16.39a	518.77 ± 16.95a	577.89 ± 22.74b
Flavonoids						
	flavonols					
	quercetin and derivatives					
**23**	Querc	1.16 ± 0.01	2.69 ± 0.11	1.76 ± 0.02	1.76 ± 0.03	1.73 ± 0.01
**24**	Querc-3-*O*-Rut	12.65 ± 0.62	1.77 ± 0.12	1.60 ± 0.04	1.73 ± 0.04	1.37 ± 0.12
**25**	Querc-3-*O*-GlucSD	60.34 ± 1.41	3.08 ± 0.19	2.46 ± 0.076	1.66 ± 0.09	1.97 ± 0.03
**26**	Querc-3-*O*-Rha	386.23 ± 0.07	33.93 ± 0.77	24.36 ± 0.91	22.62 ± 0.54	20.04 ± 0.50
**27**	Querc-Ace-GlucSD^b^	12.90 ± 0.86	0.65 ± 0.04	0.21 ± 0.01	0.18 ± 0.01	0.20 ± 0.01
**28**	Querc-3-*O*-GlucSD-7-*O*-Rha^b^	80.68 ± 0.81	19.96 ± 0.88	12.16 ± 1.03	12.85 ± 0.59	13.35 ± 0.07
**29**	Querc-3-*O*-Samb-7-*O*-Rha^b^	12.69 ± 0.80	2.32 ± 0.10	0.94 ± 0.07	0.96 ± 0.07	0.96 ± 0.01
	isorhamnetin and derivatives					
**30**	IsorhTN	tr	0.61 ± 0.06	1.11 ± 0.02	1.01 ± 0.01	0.86 ± 0.03
**31**	IsorhTN-3-*O*-GlucSD	8.16 ± 0.37	1.24 ± 0.04	2.55 ± 0.15	2.54 ± 0.19	1.91 ± 0.09
	kaempferol and derivatives					
**32**	Kmpf-MaO-GlucSD^b^	12.05 ± 0.17	0.43 ± 0.02	0.91 ± 0.09	1.10 ± 0.11	1.25 ± 0.05
	total flavonols	586.85 ± 1.89d	66.68 ± 2.16c	48.07 ± 0.14b	46.41 ± 0.81ab	43.63 ± 0.64a
	flavones					
	luteolin and derivatives					
**33**	Lut	0.15 ± 0.01	1.28 ± 0.11	0.68 ± 0.03	0.73 ± 0.01	0.78 ± 0.01
**34**	Lut-7-*O*-GlucSD	2.75 ± 0.02	0.88 ± 0.02	0.75 ± 0.00	0.80 ± 0.01	0.66 ± 0.03
**35**	Lut-8-*C*-GlucSD	36.75 ± 1.55	8.84 ± 0.72	12.84 ± 0.01	13.01 ± 0.13	11.03 ± 0.15
**36**	Lut-6-*C*-GlucSD^b^	46.57 ± 0.09	8.91 ± 0.25	13.43 ± 0.57	14.20 ± 0.42	14.45 ± 0.37
**37**	Lut-6-*C*-Hex-8-*C*-Pent^b^	6.40 ± 0.01	3.33 ± 0.12	2.95 ± 0.16	3.27 ± 0.12	3.71 ± 0.07
**38**	Lut-6-*C*-Pent-8-*C*-Hex^b^	1.71 ± 0.07	1.05 ± 0.08	0.86 ± 0.02	0.86 ± 0.03	1.24 ± 0.1
**39**	Lut-6,8-*C*-diGlucSD^b^	7.23 ± 0.05	3.91 ± 0.06	3.32 ± 0.32	3.75 ± 0.23	3.92 ± 0.0
**40**	Lut-7-*O*-(2-*O*-Ap)GlucSD^b^	38.51 ± 0.32	9.71 ± 0.28	21.01 ± 0.98	21.27 ± 1.27	21.39 ± 0.17
**41**	Lut-7-*O*-(2-*O*-Ap-Ace)GlucSD^b^	20.22 ± 1.41	1.30 ± 0.02	0.27 ± 0.02	0.42 ± 0.00	0.15 ± 0.00
**42**	Lut-7-*O*-(2-*O*-Ap-6-*O*-MaO)GlucSD^b^	699.44 ± 17.07	75.76 ± 0.35	35.13 ± 2.12	37.27 ± 1.11	40.80 ± 2.53
**43**	ChryOL-6-*C*-GlucSD^b^	1.68 ± 0.025	0.87 ± 0.01	1.58 ± 0.08	1.63 ± 0.05	2.08 ± 0.17
	apigenin and derivatives					
**44**	Apig-8-*C*-GlucSD	0.69 ± 0.02	0.15 ± 0.01	0.21 ± 0.01	0.23 ± 0.02	0.21 ± 0.02
**45**	Apig-6,8-*C*-diGlucSD	39.45 ± 0.40	22.2 ± 0.78	18.95 ± 0.71	21.47 ± 0.74	18.61 ± 0.35
**46**	Apig-Pent-Hex^b^	15.53 ± 1.26	8.44 ± 0.10	6.74 ± 0.19	7.22 ± 0.31	7.23 ± 0.43
**47**	Apig-7-*O*-(2-*O*-Ap)GlucSD^b^	5.35 ± 0.36	2.35 ± 0.11	0.65 ± 0.02	0.77 ± 0.08	0.88 ± 0.07
	total flavones	922.43 ± 14.68c	149.00 ± 2.46b	119.35 ± 3.53a	126.89 ± 0.82a	127.15 ± 3.01a
	flavanones					
	NarGE and derivatives					
**48**	NarGE	194.12 ± 5.49	2.75 ± 0.03	6.26 ± 0.29	4.19 ± 0.34	7.82 ± 0.48
**49**	NarGE-7-*O*-GlucSD	8.21 ± 0.43	0.35 ± 0.01	1.83 ± 0.09	0.82 ± 0.01	1.71 ± 0.04
	total flavanones	202.33 ± 5.29c	3.05 ± 0.12a	8.09 ± 0.33ab	5.01 ± 0.35ab	9.54 ± 0.44b
	total flavonoids	1.711.61 ± 17.41c	218.78 ± 4.44b	175.28 ± 3.45a	178.31 ± 1.76a	180.53 ± 3.99a
	total phenolic compounds	2.736.34 ± 9.49d	1.099.38 ± 18.53c	684.054 ± 19.34a	696.88 ± 18.32a	756.66 ± 22.95b

a Results are expressed as μg of (poly)phenolic compounds per g of pepper (dry matter) (mean ± standard deviation, *n* = 3).

b Tentatively identified compounds.

c Full compound names are shown in Table S1. Different letters for each row indicate significant differences (*p* ≤ 0.05) among samples.

Abbreviations: nd=not detected; tr=traces.

A similar (poly)phenolic profile has been previously reported for raw pepper, highlighting flavonoids as predominant compounds, specifically quercetin, luteolin, and their glycoside derivatives.^5,15,28,34,35^ In line with our results, cinnamic acids have been also described as highly present compounds among phenolic acids in raw pepper, including caffeic acid (3′,4′-dihydroxycinnamic acid), ferulic acid (4′-hydroxy-3′-methoxycinnamic acid), *p*-coumaric acid (4′-hydroxycinnamic acid), and their respective glycosides.^35−37^ Isorhamnetin 3-*O*-glucoside, which has been suggested to have an anti-inflammatory effect and to prevent oxidative stress and lipid peroxidation,^38,39^ has not been previously identified in *C. annuum*. Moreover, to our best knowledge, isorhamnetin and 2′-hydroxy-4′-methoxyacetophenone, previously detected in other pepper species (*C. baccatum*),^3^ have been detected for the first time in *C. annuum* species. Although expected to be present, other (poly)phenolic compounds, including vanillic acid (4-hydroxy-3-methoxybenzoic acid), vanillin (4-hydroxy-3-methoxybenzaldehyde), 4-hydroxybenzoic acid, apigenin, myricetin, and kaempferol-*O*-glucoside, identified in other *C. annuum* varieties,^4,6,7,40^ were not detected in raw Piquillo pepper samples.

Regarding the (poly)phenolic content of raw Piquillo pepper, flavonoids were the most abundant compounds quantified, accounting for 62.6% (1711.61 μg/g of pepper dry matter, dm) of the total (poly)phenolic content (2736.34 μg/g dm), whereas nonflavonoids represented 37.4% (1024.73 μg/g dm). In previous studies, quercetin-3-*O*-rhamnoside has been reported as the predominant (poly)phenolic compound in raw pepper.^15,16,35,37^ However, in the present study luteolin-7-*O*-(2-*O*-apiosyl-6-*O*-malonyl)glucoside (compound **42**) and cinnamic-4′-*O*-glucoside (compound **9**) were the most abundant compounds in raw Piquillo pepper, accounting for 25.6 and 16.4% of the total (poly)phenolic content, respectively, and showed higher concentrations (699.4 and 477.8 μg/g dm) than quercetin 3-*O*-rhamnoside (386.2 μg/g dm) (compound **26**). Luteolin-*O*-(apiosyl-malonyl)glucoside has also been stated as the major compound in red pepper cv. *Capia*. Quercetin 3-*O*-rhamnoside was apparently not detected in this study, but it is uncertain whether it was not found or not sought for.^5^ Jeong et al.^36^ have also described luteolin derivatives as predominant compounds in two bell pepper varieties (*C. annuum* L. red, cv. *Cupra* and orange, *cv. Orange glory*), whereas in white *cv. ST4712* pepper, quercetin derivatives have been reported as predominant compounds, explained by the greater content of quercetin 3-*O*-rhamnoside.

The (poly)phenolic content of pepper previously reported in the literature is highly variable since it strongly depends on variety, maturation state, and country of origin, as well as the extraction procedures and quantification assays performed.^2,6,7,34^ Moreover, as previously stated, quantitative analysis on individual (poly)phenolic compounds has not been extensively performed in pepper, with the additional problem that for some flavonoids conjugates, which have been suggested to be present in high quantities, no pure standards were available, and these compounds have been commonly quantified using equivalents, which might overestimate or underestimate their real content in the current work and in those from other research groups.^41^ Furthermore, concentrations are not equally expressed (fresh or dry matter), making it difficult to compare published results among different pepper varieties or other plant-based foods. For this reason, the (poly)phenolic content is discussed on the basis of dry matter, given that pepper has on average approximately 90% of water.^42,43^ Total (poly)phenolic content determined in raw Piquillo pepper (2736.34 μg/g dm, Table 1) was in line with previous concentrations found in raw *cv. Aleppo* (*C. annuum*) by Kelebek et al.^5^ (2457.1 μg/g dm) and in red, *cv. Cupra*, and orange, *cv. Orange glory* pepper (2856 and 3167 μg/g dm respectively).^36^ Immature green *cv. Vergasa* sweet pepper also presented similar (poly)phenols concentrations (2223 μg/g dm).^34^ However, (poly)phenolic reduction was observed after the three stages of maturation studied, green, immature red, and fully mature red (431, 361, and 298 μg/g dm, respectively).^34^ Moreover, important differences have been found in other *C. annuum* varieties. On the one hand, significantly lower content has been reported in raw *C. annuumcv. Capia* (540 μg/g dm) Italian green pepper (360 μg/g dm) and red *Chiltepin cv. glabriusculum* (452.9 μg/g dm),^5,15,44^ whereas in contrast, white *cv. ST4712* (*C. annuum*) presented a considerably higher amount of the total (poly)phenolic content (20,137 μg/g dm), mainly due to the high concentrations of quercetin 3-*O*-rhamnoside found (15,018 μg/g dm).^36^

### 3.2 Impact of Heat Treatment on Total and Individual (Poly)phenolic Compounds

#### 3.2.1 Industrial Heat Treatments

In accordance with the regulations established by the PDO, Piquillo pepper is submitted to industrial grilling followed by peeling and a canning process before commercialization. Both thermal processes are characterized by using high temperatures, and therefore, effects on total and individual (poly)phenolic compounds were foreseen.^17,45^ Moreover, peel has been suggested to contain high amounts of flavonoids conjugates and cinnamic acid derivatives,^34^ and in consequence, its removal was also expected to influence the total (poly)phenolic content after industrial processing.

**Figure 1:**
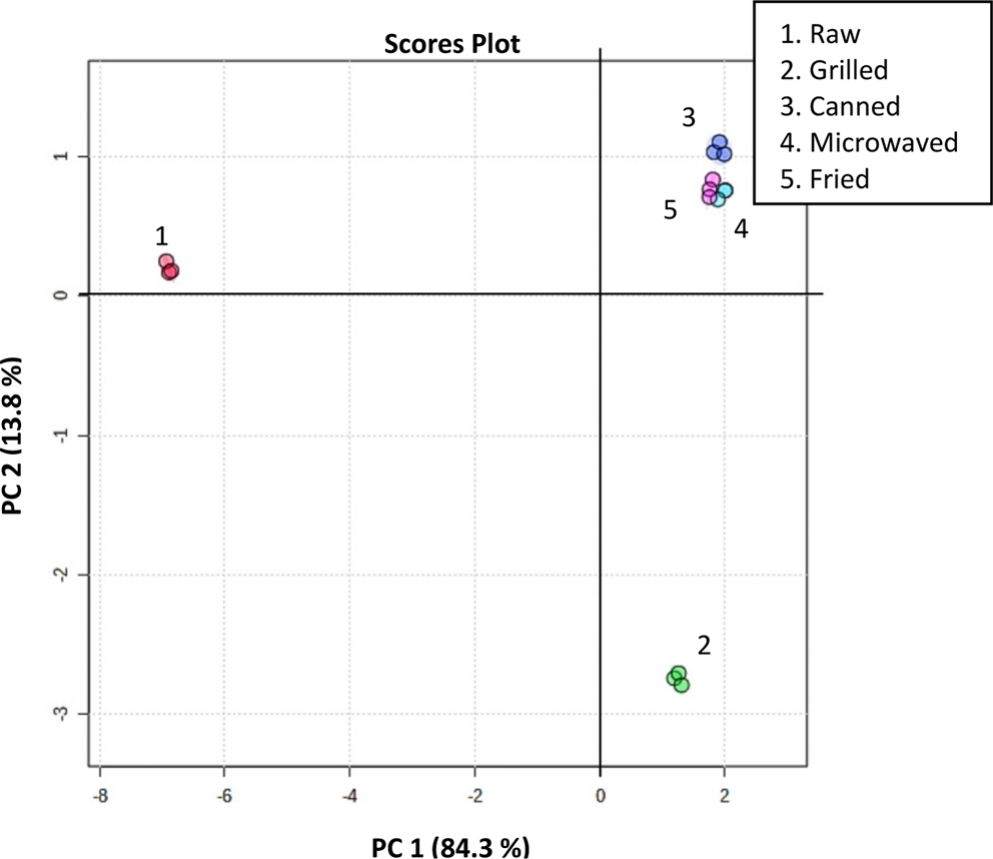
Principal Component Analysis (PCA) of the (poly)phenolic contents of Piquillo pepper before and after industrial (grilled and canned) and culinary (microwaved and fried) heat treatments. The explained variances are shown in brackets.

PCA performed clearly displays the distribution of raw and thermally treated samples and shows how the thermal treatments applied, especially industrial heat treatments, influence the total (poly)phenolic content of Piquillo pepper (Figure 1). This statistical procedure generated a two-component model that accounted for 98.1% of the total variance, where the PC1 explained 84.3% and PC2 13.8%. Raw Piquillo pepper samples are grouped on the left upper side of the graphic (Figure 1), whereas all thermally treated samples are diametrically opposed on PC1 axis, confirming the impact of food processing on (poly)phenolic compounds. Interestingly, grilled Piquillo peppers are also opposed on PC2 axis when compared to raw peppers, highlighting the strong variance between both samples.

**Figure 2:**
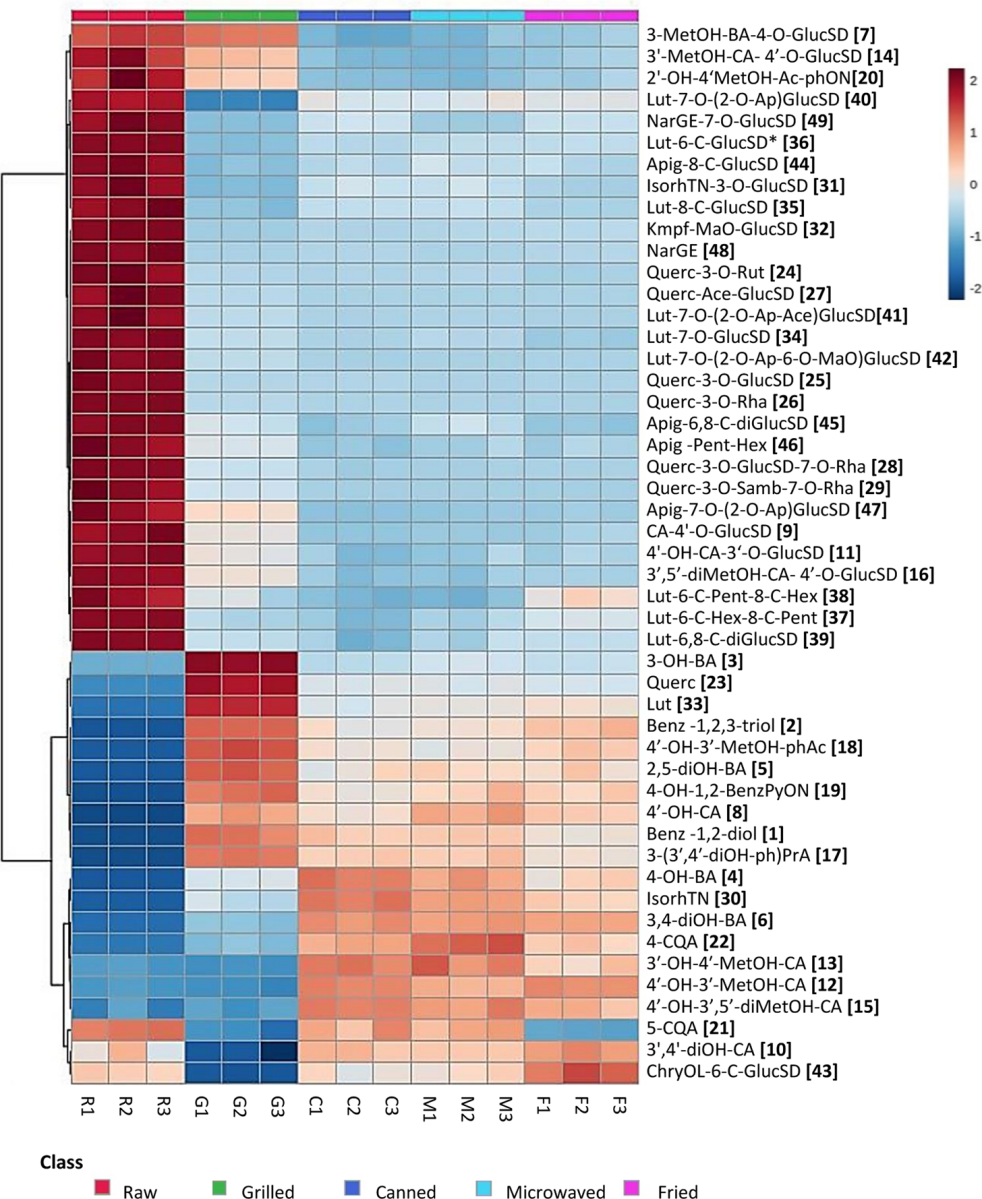
Heatmap of (poly)phenolic profile in Piquillo pepper before and after industrial (grilled and canned) and culinary (microwaved and fried) heat treatments. Compounds were hierarchically clustered according to the Ward clustering method and based on Euclidean distance. Full compound names are shown in Table S1.

In this sense, the extremely high temperatures applied during grilling, even for a short period of time, together with the subsequent peeling process, impact not only the total content but also the individual (poly)phenolic compounds of Piquillo pepper. Overall, total (poly)phenolic compounds significantly decreased after grilling from 2736.34 to 1099.38 μg/g dm (59.8% reduction) (Table 1). In particular, flavonoids showed a higher reduction of 87.2% after grilling compared to nonflavonoids, which only decreased by 14%. The variation rate of each individual compound is represented in Figure 2, with colors ranging from dark blue (strong reduction) to dark red (strong increase), and as can be clearly observed, not all compounds were equally affected by heat treatments, even within (poly)phenolic families or subgroups. Thus, the effect of heat treatments may depend not only on the molecular structure of the (poly)phenols family but also on the individual structure of each compound.

On the one hand, flavonoid glycosides were strongly reduced after grilling, whereas quercetin and luteolin aglycones (compounds **23** and **33**) greatly increased, probably due to the thermal breakdown of chemical bonds and degradation of their respective glycoside complexes^19,46^ (Figure 2). Moreover, industrial grilling includes a peeling process, which might partly contribute to (poly)phenols reduction observed in Piquillo pepper since the peel of several fruits and vegetables has been reported to contain high amounts of (poly)phenols, especially flavonoid conjugates.^34,47,48^ Regarding nonflavonoids, cinnamic acid glucosides, including cinnamic-4′-*O*-glucoside (coumaric acid glucoside, compound **9**), 4′-hydroxycinnamic-3-*O*-glucoside (caffeic acid glucoside, compound **11**), 3′-methoxycinnamic-4′-*O*-glucoside (ferulic acid glucoside, compound **14**), and 3′,5′-dimethoxycinnamic-4′-*O*-glucoside (sinapic acid glucoside, compound **16**) were also thermally degraded during grilling, although to a lesser extent, suggesting that nonflavonoid glycosides were either more thermostable or less abundant in peel than the flavonoid conjugates (Figure 2). Interestingly, 3′methoxybenzoic acid 4-glucoside (vanillic acid glucoside, compound **7**) was not greatly reduced by grilling compared to the previously mentioned cinnamic acid glucosides (Figure 2), highlighting the significance of the chemical structure on (poly)phenolic thermostability. Besides, for (poly)phenol glycosides degradation, total (poly)phenolic reduction might be also explained by their incorporation into melanoidins formed by Maillard reactions, occurring during grilling as a result of the high temperatures applied.^19,28^

On the other side, Figure 2 clearly illustrates how 8 nonflavonoids greatly increased after grilling, including 2 benzenediols and -triols (compounds **1** and **2**) 2 benzoic acids (compounds **3** and **5**), 1 phenylpropanoic acid (compound **17**), 1 phenylacetic (compound **18**), and 1 nonflavonoid (compound **19**) classified as others. More specifically, these compounds were not detected in raw Piquillo pepper (Table 1), suggesting that although (poly)phenols reduction might be partly explained by the peeling process, flavonoid conjugates were also thermally degraded, resulting in other lower molecular weight (poly)phenols. The appearance of three hydroxy- and dihydroxybenzoic acids (compounds **3**, **5**, and **6**) after grilling, which are generally attached to cell wall polysaccharides, supported the hypothesis that thermal treatment applied to vegetables lead to the hydrolysis of polysaccharide complexes and therefore increase the extractability of some (poly)phenolic compounds.^16,46,49^ Compounds **3** and **18** (3-hydroxybenzoic and 4′-hydroxy-3′-methoxyphenylacetic acid) have been also identified in thermally treated pepper but only in *C. baccatum* species.^3^ Moreover, three (poly)phenolic compounds, detected only after grilling, (benzene-1,2-diol, 2,5-dihydroxybenzoic acid, and 3-(3′,4′-dihydroxyphenyl)propanoic acid) (compounds **1**, **5**, and **17** respectively) were found for the first time in pepper samples. Juaniz et al.^28^ detected 3-(3′,4′-dihydroxyphenyl)propanoic acid but after the action of gut microbiota during in vitro colonic fermentation of green pepper, but neither in raw or heat-treated green pepper nor after the action of enzymes and acids during gastrointestinal digestion, suggesting that this phenolic acid resulted from the colonic catabolism of other native (poly)phenolic compounds present in Italian green pepper (*C. annuum*).

Flavonoids decrease, and the appearance of new lower molecular weight compounds, in particular phenolic acids, after grilling, resulted in the modification of the (poly)phenolic profile of Piquillo pepper. In raw samples, phenolic acids accounted for 37.4% and flavonoids for 62.6%, whereas in grilled pepper, phenolic acids became the predominant compounds (80.1%), and only 19.9% corresponded to the flavonoid content. Other authors have also reported phenolic acids as the most abundant compounds in thermally treated pepper samples.^7,44^ However, no data regarding the (poly)phenolic content of raw samples were available, and therefore, it is uncertain if there was an effect of heat treatments. Moreover, among flavonoids, only aglycones were studied, and as stated by several authors, flavonoids naturally occur as glycosides in plant-based foods.^7,17,46^

Successive industrial canning processes after grilling further significantly reduced total (poly)phenolic content from 1099.38 to 684.05 μg/g dm (37.8% reduction). In the distributional representation of PCA (Figure 1), canned pepper samples are located in the upper right graph, opposed on the PC2 axis when compared to grilled peppers. In particular, an additional decrease after canning was observed for cinnamic acid glycosides as well as for flavonoid glycosides. Moreover, the eight nonflavonoids that appeared after grilling (compounds **1**, **2**, **3**, **5**, **8**, **17**, **18**, and **19**) as well as quercetin and luteolin aglycones (compounds **23** and **33**) were also thermally degraded during canning (Figure 2). On the contrary, some (poly)phenols (compounds **4**, **6**, **12**, **13**, **15**, **22**, and **30**) that were hardly affected by grilling, presented an increase after canning, probably explained by the degradation of their respective glycosides or by the chemical transformation of other phenols due to the thermal breakdown of bound molecules. In particular, 4-hydroxybenzoic acid (compound **4**) and 3,4-dihydroxybenzoic acid (compound **6**), appearing after grilling, showed a notable increase after canning, probably due to the reduction of 3-methoxybenzoic-4-glucoside (compound **7**), observed only after canning (Table 1). Nevertheless, changes in polyphenol content and profile were less marked than after grilling (Figure 2), suggesting that temperature (ca. 700 °C during grilling vs 102 °C during canning) together with peeling were the most important parameters of food processing affecting (poly)phenols rather than heating time (15 s vs 30 min). Despite canning being a widely used method for food preservation, little information is available regarding its influence on (poly)phenolic content, and to the best of our knowledge, no studies have evaluated the impact on *C. annuum*. Le Bourvellec et al.^50^ and Parmar et al.,^51^ have also reported a decrease in total (poly)phenolic content in apricots (*Prunus armeniaca* L.) and legume grains (kidney bean, chickpea, and field pea) after industrial canning processes, probably due to the thermal degradation and leakage of soluble phenolic acids to the surrounding medium.

Overall, to our best knowledge, this is the first time that the effect of industrial heat treatments on pepper (poly)phenols was evaluated, especially at temperatures higher than 200–220 °C. As stated by Eyarkai Nambi et al.,^52^ (poly)phenolic reduction rate is logarithmic at high temperatures, even for short periods of cooking time, which in combination with peeling process, might explain the strong reduction of total (poly)phenols and especially of flavonoid glycosides after grilling (ca. 700 °C). Nonflavonoid glycosides appeared to be more thermostable, and moreover, some low molecular weight compounds were generated as result of thermal processing. In this sense, it should be stressed out that once ingested, (poly)phenolic compounds have been reported to be poorly absorbed in the human body since they are commonly found glycosylated or bound to other molecules, forming complexes.^45^ Therefore, (poly)phenols naturally found in plant-based foods need to be hydrolyzed into aglycones or lower molecular weight compounds by intestinal enzymes or gastrointestinal conditions, or released from the food matrix to become available for their absorption (bioaccessibility) and further reach the target cells or organs (bioavailability) for having the potential health effects attributed (bioactivity).^17,45,46^ For this reason, the chemical transformation of (poly)phenolic compounds from Piquillo pepper into lower molecular weight compounds and the physical modification (cell wall softening) resulting mainly from industrial processing, might enhance their bioaccessibility, favoring their absorption and eventually, their bioactivity.

#### 3.2.2 Culinary Heat Treatments

The PCA graph further illustrates the much less marked impact of culinary processed peppers (microwaved and fried) on the (poly)phenolic profile when compared to canned Piquillo pepper (Figure 1). Specifically, the total (poly)phenolic content of microwaved and fried peppers (696.88 and 756.66 μg/g dm, respectively) barely differed from canned Piquillo pepper (684.05 μg/g dm) since both processing time and temperature applied during culinary treatments were much lower than for industrial heat treatments. In the case of microwaved Piquillo pepper, the short time (1 min) and lower temperature reached compared to industrial processing or frying did not result in substantial changes regarding both, the total content and (poly)phenolic profile of canned Piquillo pepper. Similarly, Domínguez-Fernández et al.^33^ reported no differences on the total (poly)phenolic content of artichokes after microwaving besides power and time applied, which were slightly higher than in the present study (900 W for 4 min vs 750 W for 1 min).

However, after frying, a slight but significant increase in total (poly)phenolic compounds was observed compared to canned pepper (10.6% increase, Table 1), mainly due to the increase of nonflavonoids, in particular cinnamic and benzoic acids. It could be hypothesized that this increase may be at least partially associated with the addition of olive oil during frying. However, phenolic compounds previously characterized in olive oil, including hydroxytyrosol, tyrosol, or oleuropein were not detected in fried pepper samples. This could be probably explained by the low amount of oil added (15 mL) and also because it was a nonvirgin olive oil, mainly composed of refined olive oil and a low quantity of virgin olive oil.^53^ Therefore, it can be suggested that the use of lower temperatures compared to industrial processing, and the short period of time during culinary processes did not result in the further degradation of (poly)phenolic compounds. On the contrary, it leads to physical modifications of the food matrix and cell wall softening that seem to increase (poly)phenol release and extractability, especially after frying.^17,19^

In general, some authors have observed a substantial (poly)phenols increase in pepper after culinary treatments, indicating that thermal breakdown of cell walls favors the release of bound phenolic compounds and phenolic extractability from food matrix.^5,15,22,24^ Contrary to our results, increases in total (poly)phenolic content, measured by Folin–Ciocalteu, were observed after the roasting process at 90 °C for 25 min in chili peppers^22^ and in Jalapeño pepper after oven drying at 60 °C for 36 h.^24^ Similarly, Ornelas-Paz et al.^27^ evaluated the effect of boiling (96 °C for 7–13.5 min) and grilling (210 °C for 8.8–19 min) on the total (poly)phenols of several Mexican pepper varieties revealing, in general, an increase (7.4–137%) of these compounds with respect to raw pepper, especially after grilling, but changes were greatly dependent on pepper variety. In particular, like the results observed in the present research, the total (poly)phenolic content of sweet bell pepper (green, yellow, and red) was reduced after cooking (1.6–26.9%).^27^ Therefore, it might be suggested that different behavior on the total (poly)phenolic content after the application of thermal treatments might also be modulated by other pepper components (content in vitamins, carotenoids, capsaicinoids, etc.). Moreover, it should be considered that the use of a nonspecific quantification assay (Folin–Ciocalteu) might mislead the effect of thermal treatments since other compounds (for instance, vitamin C or carbonyls derived from Maillard reactions) might also have a reducing activity.^19^ In addition, this assay does not allow evaluating the effect on individual (poly)phenolic compounds. In this context, only two studies that evaluate (poly)phenols by LC–MS have been found. Kelebek et al.^5^ also reported flavonoid conjugates as the most abundant compounds quantified by LC–MS in two pepper varieties (*Capia* and *Aleppo* peppers) which, contrary to our results, increased after the application of heat processing (220 °C for 5 min). On the other hand, and similar to our results, Juániz et al.^28^ found a reduction in total (poly)phenolic compounds after different cooking processes in Italian green pepper. Moreover, a lower decrease of total (poly)phenols after grilling (9.4%) than after frying (62.8%)^28^ indicated that time and temperature applied during processing also impact the (poly)phenols of pepper differently. Differences with results observed in the present work might be partly explained because full pepper fruits, including peel, were analyzed in both studies, whereas in Piquillo pepper, the peel was removed after grilling. Therefore, the existing evidence concerning whether thermal treatment decreases or increases total (poly)phenolic content in *C. annuum* is contradictory among researches since it might depend on the food matrix (variety, whole pepper vs peeled pepper, etc.), and more specifically, on thermal treatment conditions along with the methodology applied for the extraction and determination of the (poly)phenolic content.^19^

In conclusion, an extensive characterization of (poly)phenolic compounds present in *C. annuum* was performed by using a high number of pure phenolic standards, besides evaluating the effect of industrial and culinary treatments on individual and total (poly)phenolic composition. Overall, high temperatures applied during industrial grilling and subsequent peeling produced a strong reduction of total (poly)phenolic content, especially of flavonoids, the predominant compounds in raw Piquillo pepper (62.2%). Moreover, the generation of nine additional phenolic acids after grilling, probably derived from the thermal degradation of other (poly)phenols or their release from food matrix, modifies the (poly)phenolic profile of Piquillo pepper, making phenolic acids the most abundant compounds in heat-treated samples (74.4–80.1%). Subsequent canning processes reduced the total (poly)phenolic content mainly due to the cleavage of cinnamic and benzoic acid glycosides, whereas in the case of subsequent culinary treatments (microwaving and frying), (poly)phenolic content was not notably affected. Therefore, it is suggested that although the grilling technique strongly degrades (poly)phenolic compounds, it also generates low-molecular-weight compounds that might be more efficiently absorbed than those present in raw pepper. Moreover, subsequent heat treatments at moderately high temperatures might favor the release of (poly)phenolic compounds from complexes within the food matrix during digestion and might enhance their bioaccessibility and eventually their absorption and bioavailability. Future research on the bioaccessibility and colonic biotransformation of (poly)phenolic compounds present in Piquillo pepper is of great interest and should be conducted in order to clarify their potential health implications.

## Supporting Information Available

Summary of the (poly)phenolic compounds identified and quantified in Piquillo pepper named according to the recommended standardizing nomenclature proposed by Kay et al.^30^ and mass spectrometric identification parameters of (poly)phenolic compounds identified in Piquillo pepper determined by LC–MS/MS (PDF)

## Abbreviations

HPLC: high-performance liquid chromatography
MS/MS: mass spectrometry
LC: liquid chromatography
PDO: protected designation of origin
CE: collision energy
dm: dry matter
PCA: principal component analysis
PC: principal component.
