# Supplementary material for: LC–MS/MS Analysis Elucidates the Different Effects of Industrial and Culinary Processing on Total and Individual (Poly)phenolic Compounds of Piquillo Pepper (Capsicum annuum cv. Piquillo)
Source: J Agric Food Chem. 2023 Apr 4;71(15):6050–60. doi: 10.1021/acs.jafc.2c07829 (PMC10119983; doi:10.1021/acs.jafc.2c07829)
Supplement: Supplementary file 1 — jf2c07829_si_001.pdf [file jf2c07829_si_001.pdf]

SUPPORTING INFORMATION

LC-MS/MS Analysis Elucidates the Different Effects of Industrial and Culinary Processing on Total and Individual (Poly)phenolic Compounds of Piquillo Pepper (*Capsicum annuum cv.* Piquillo)

Cristina Del Burgo-Gutiérrez[1,2], Concepción Cid[1,2,3], Iziar A. Ludwig[1,2,3*] and María-Paz De Peña[1,2,3].

[1] University of Navarra, Faculty of Pharmacy & Nutrition, Department of Nutrition Food Science & Physiology, 31008 Pamplona, Spain

[2] University of Navarra, Center for Nutrition Research, c/Irunlarrea 1, 31008 Pamplona, Spain

[3] IdiSNA, Navarra Institute for Health Research, 31008 Pamplona, Spain

*Corresponding author: Iziar A. Ludwig. *E-mail address: iludwig@unav.es Tel: +34 948425600 (ext. 806652)

Table S1. Summary of (poly)phenolic compounds identified and quantified in Piquillo pepper named according to the recommended standardizing nomenclature proposed by Kay et al. [30].

	Recommended name	Other common names	Abbreviation
	**NONFLAVONOIDS**		
	**Benzenediols and triols**	**Benzenes**	
**1**	Benzene -1,2-diol	1,2-Dihydroxybenzene, Catechol	Benz-1,2-diol
**2**	Benzene -1,2,3-triol	1,2,3-Trihydroxybenzene, Pyrogallol	Benz-1,2,3-triol
	**Benzoic acids**	**Hydroxybenzoic acids**	
**3**	3-Hydroxybenzoic acid	-	3-OH-BA
**4**	4-Hydroxybenzoic acid	-	4-OH-BA
**5**	2,5-Dihydroxybenzoic acid	-	2,5-diOH-BA
**6**	3,4-Dihydroxybenzoic acid	Protocatechuic acid	3,4-diOH-BA
**7**	3-Methoxybenzoic acid 4-O-glucoside	Vanillic acid glucoside	3-MetOH-BA-4-O-GlucSD
	**Cinnamic acids**	**Hydroxycinnamic acids**	
**8**	4’-Hydroxycinnamic acid	p-coumaric acid	4'-OH-CA
**9**	Cinnamic-4'-O-glucoside	Coumaric acid glucoside	CA-4'-O-GlucSD
**10**	3',4'-Dihydroxycinnamic acid	Caffeic acid	3',4'-diOH-CA
**11**	4’-Hydroxycinnamic-3-O-glucoside	Caffeic acid glucoside	4'-OH-CA- 3'-O-GlucSD
**12**	4’-Hydroxy-3’-methoxycinnamic acid	Ferulic acid	4'-OH-3'-MetOH-CA
**13**	3’-Hydroxy-4’-methoxycinnamic acid	Isoferulic acid	3'-OH-4'-MetOH-CA
**14**	3’-Methoxycinnamic-4’-O-glucoside	Ferulic acid glucoside	3'-MetOH-CA-4’-O-GlucSD
**15**	4’-Hydroxy-3’,5’-dimethoxycinnamic	Sinapic acid	4'-OH-3',5’-diMetOH-CA
**16**	3’,5’-Dimethoxycinnamic-4’-O- glucoside	Sinapic acid glucoside	3',5’-diMetOH-CA-4’-O-GlucSD
	**Phenylpropanoic acids**	**Phenylpropionic acids**	
**17**	3-(3’,4’-Dihydroxyphenyl)propanoic acid	Dihydrocaffeic acid	3-(3',4'-diOH-ph)-PrA
	**Phenylacetic acids**		
**18**	4’-Hydroxy-3’-methoxyphenylacetic acid	Homovanillic acid	4'-OH-3'-MetOH-phAc
	**Others**		
**19**	4-Hydroxy-1,2-benzopyrone	4-Hydroxycoumarin	4-OH-1,2-BenzPyON
**20**	2'-Hydroxy-4'-methoxyacetophenone	Paeonol	2'-OH-4'MetOH-Ac-phON
	**Acyl-quinic acids**	**Chlorogenic acids**	
**21**	5- Caffeoylquinic acid	Chlorogenic acid	5-CQA
**21**	4- Caffeoylquinic acid	Cryptochlorogenic acid	4-CQA
	**FLAVONOIDS**		
	**Flavonols**		
	-Quercetin and derivatives		
**23**	Quercetin	Quercetin	Querc
**24**	Quercetin-3-O-rutinoside	Rutin	Querc-3-O-Rut
**25**	Quercetin-3-O-glucoside	Isoquercitrin	Querc-3-O-GlucSD
**26**	Quercetin-3-O-rhamnoside	Quercitrin	Querc-3-O-Rha
**27**	Quercetin-acetyl-glucoside	-	Querc-Ace-GlucSD
**28**	Quercetin-3-O-glucoside-7-O-rhamnoside	-	Querc-3-O-GlucSD-7-O-Rha
**29**	Quercetin-3-O-sambubioside-7-O-rhamnoside	-	Querc-3-O-Samb-7-O-Rha

Table S1. (Continued)

	Recommended name	Other common names	Abbreviation
	- Isorhamnetin and derivatives		
**30**	Isorhamnetin	-	IsorhTN
**31**	Isorhamnetin-3-O-glucoside	-	IsorhTN-3-O-GlucSD
	- Kaempferol and derivatives		
**32**	Kaempferol-malonyl-glucoside	-	Kmpf-MaO-GlucSD
	**Flavones**		
	- Luteolin and derivatives		
**33**	Luteolin	-	Lut
**34**	Luteolin-7-O-glucoside	-	Lut-7-O-GlucSD
**35**	Luteolin-8-C-glucoside	Orientin	Lut-8-C-GlucSD
**36**	Luteolin-6-C-glucoside	Isoorientin	Lut-6-C-GlucSD
**37**	Luteolin-6-C-hexoside-8-C-pentoside	-	Lut-6-C-Hex-8-C-Pent
**38**	Luteolin-6-C-pentoside-8-C-hexoside	-	Lut-6-C-Pent-8-C-Hex
**39**	Luteolin-6,8-C-diglucoside	Lucenin-2	Lut-6,8-C-diGlucSD
**40**	Luteolin-7-O-(2-O-apiosyl)glucoside	-	Lut-7-O-(2-O-Ap)GlucSD
**41**	Luteolin-7-O-(2-O-apiosylacetyl)glucoside	-	Lut-7-O-(2-O-Ap-Ace)GlucSD
**42**	Luteolin-7-O-(2-O-apiosyl-6-O-malonyl)glucoside	-	Lut-7-O-(2-O-Ap-6-O-MaO)GlucSD
**43**	Chrysoeriol 6-C-glucoside	Isoscoparin	ChryOL 6-C-GlucSD
	- Apigenin and derivatives		
**44**	Apigenin-8-C-glucoside	Vitexin	Apig-8-C-GlucSD
**45**	Apigenin-6,8-C-diglucoside	Vicenin-2	Apig-6,8-C-diGlucSD
**46**	Apigenin-pentoside-hexoside	-	Apig-Pent-Hex
**47**	Apigenin-7-O-(2-O-Apiosyl)glucoside	Apiin	Apig-7-O-(2-O-Ap)GlucSD
	**Flavanones**	**Flavanones**	**Flavanones**
	- Naringenin and derivatives		
**48**	Naringenin	-	NarGE
**49**	Naringenin-7-O-Glucoside	-	NarGE-7-O-GlucSD

Table S2. Mass spectrometric identification parameters of (poly)phenolic compounds identified in Piquillo pepper determined by LC-MS/MS.

	Compound	Rt	$[M-H]^-$ (m/z)	MS/MS Fragmentation	CE (eV)
	**NONFLAVONOIDS**				
	**Benzenediols and triols**				
**1**	Benz -1,2-diol	3.15	109	108, 91	-25
**2**	Benz-1,2,3-triol*	1.30	125	79, 81, 97	-25
	**Benzoic acids**				
**3**	3-OH-BA	6.24	137	93,109	-20
**4**	4-OH-BA	4.12	137	93,001	-15
**5**	2,5-diOH-BA	4.40	153	108, 109	-30
**6**	3,4-diOH-BA	2.20	153	109, 91, 98	-35
**7**	3-MetOH-BA-4-O-GlucSD*	3.10	329	167, 209	-25
	**Cinnamic acids**				
**8**	4’-OH-CA	8.70	163	119, 93	-30
**9**	CA-4'-O-GlucSD*	5.80	325	163, 119, 117	-25
**10**	3',4'-diOH-CA	6.31	179	135, 134	-30
**11**	4'-OH-CA-3'-O-GlucSD*	6.20	341	179, 135	-20
**12**	4’-OH-3’-MetOH-CA	9.45	193	134, 178	-20
**13**	3’-OH-4’-MetOH-CA	9.50	193	178, 134	-15
**14**	3'-MetOH-CA- 4’-O-GlucSD*	7.10	355	175, 193, 295	-20
**15**	4’-OH-3’,5’-diMetOH-CA	9.60	223	193, 121	-30
**16**	3’,5’-diMetOH-CA- 4’-O-GlucSD*	7.50	385	223, 164	-25
	**Phenylpropanoic acids**				
**17**	3-(3’,4’-diOH-ph)PrA	5.60	181	59, 137	-25
	**Phenyalacetic acids**				
**18**	4’-OH-3’-MetOH-phAc*	7.30	181	137, 163, 122	-25
	**Other phenolic acids**				
**19**	4-OH-1,2-BenzPyON*	6.50	161	117, 133	-30
**20**	2'-OH-4'MetOH-Ac-phON*	8.70	165	150	-25
	**Chlorogenic acids**				
**21**	5-CQA	5.85	353	191. 179	-20
**22**	4-CQA	6.73	353	173, 179	-30
	**FLAVONOIDS**				
	**Flavonols**				
	- Quercetin and derivatives				
**23**	Querc	15.92	301	151, 179	-30
**24**	Querc-3-O-Rut	9.96	609	301, 179	-50
**25**	Querc-3-O-GlucSD	10.25	463	300, 301, 271	-35
**26**	Querc-3-O-Rha	12.28	447	301, 283	-40
**27**	Querc-Ace-GlucSD*	11.50	505	300, 271	-30
**28**	Querc-3-O-GlucSD-7-O-Rha*	8.30	609	301, 463, 447	-40
**29**	Querc-3-O-Samb-7-O-Rha*	8.4	741	299, 301, 270	-65

Table S2. (Continued)

	Compound	Rt	[M-H]⁻ (m/z)	MS/MS Fragmentation	CE (eV)
	- Isorhamnetin and derivatives				
**30**	IsorhTN	18.30	315	300, 151	-30
**31**	IsorhTN-3-O-glucoside	12.49	477	314, 271, 285	-25
	- Kaempferol and derivatives				
**32**	Kmpf-MaO-GlucSD*	13.22	533	489, 285	-30
	**Flavones**				
	- Luteolin and derivatives				
**33**	Lut	15.83	285	133, 151	-50
**34**	Lut-7-O-GlucSD	10.32	447	285, 327	-30
**35**	Lut-8-C-GlucSD	9.38	447	327,297,285	-35
**36**	Lut-6-C-GlucSD*	9.18	447	327, 257, 297, 285	-25
**37**	Lut-6-C-Hex-8-C-Pent*	8.50	579	459, 489	-30
**38**	Lut-6-C-Pent-8-C-Hex*	8.90	579	489, 459	-30
**39**	Lut-6,8-C-diGlucSD*	8.10	609	369, 399, 489, 519	-55
**40**	Lut-7-O-(2-O-Ap)GlucSD*	10.26	579	285, 447, 327	-40
**41**	Lut-7-O-(2-O-Ap-Ace)GlucSD*	13.01	621	285, 489, 579, 327	-55
**42**	Lut-7-O-(2-O-Ap-6-O-MaO)GlucSD*	13.20	665	621, 285, 489, 579, 561	-40
**43**	ChryOL-6-C-GlucSD*	10.44	461	341, 371, 299	-30
	- Apigenin and derivatives				
**44**	Apig-8-C-GlucSD	9.94	431	311, 283	-30
**45**	Apig-6,8-C-diGlucSD	8.56	593	353, 383, 473	-45
**46**	Apig-Pent-Hex *	8.98	563	383, 473, 443	-40
**47**	Apig-7-O-(2-O-Ap)GlucSD *	16.20	563	131, 151, 117	-40
	**Flavanones**				
	- Naringenin and derivatives	12.56	431	269, 311, 283	-40
**48**	NarGE	17.90	271	151, 177, 118, 93	-20
**49**	NarGE-7-O-glucoside	12.51	433	271, 313	-20

Rt, retention time; m/z, mass-to-charge ratio; [M–H]⁻, Negatively charged molecular ion; MS^2, MS/MS fragmentation; CE, Collision energy. *=Tentatively identified compounds.
